# Supplementary material for: The interactome of intact mitochondria by cross-linking mass spectrometry provides evidence for coexisting respiratory supercomplexes
Source: Mol Cell Proteomics. 2017 Dec 8;17(2):216–32. doi: 10.1074/mcp.RA117.000470 (PMC5795388; doi:10.1074/mcp.RA117.000470)
Supplement: Supplemental Data [file supp_RA117.000470_133922_0_supp_23978_fzffwf.zip › spectra_annotation/mito_DR_spectra_annotation/309-1-32-1-7-1.pdf]

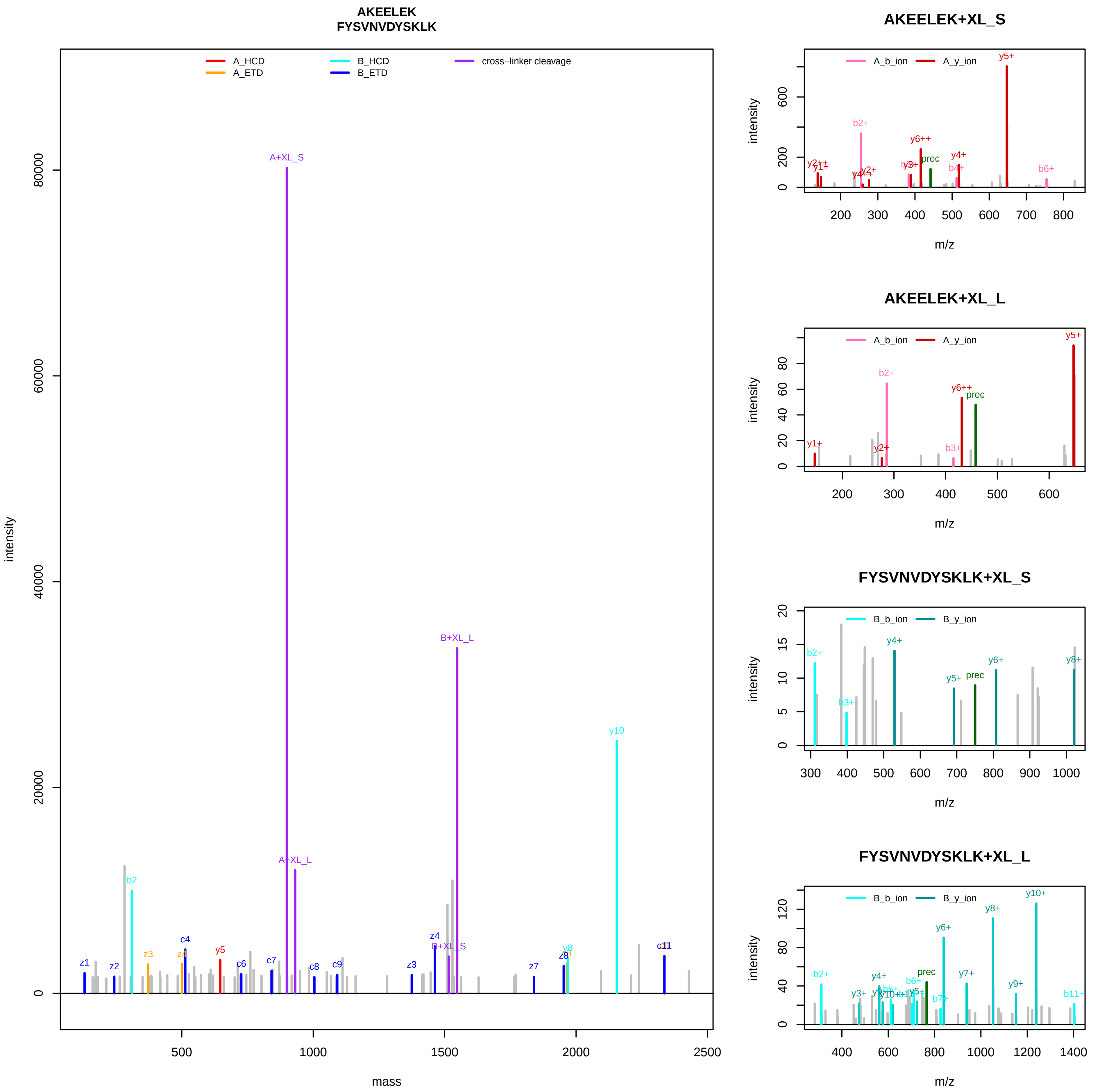

AKEELEK
FYSVNVDYSKLK
A_HCD
A_ETD
B_HCD
B_ETD
cross−linker cleavage
A+XL_S
B+XL_L
A+XL_L
B+XL_S
y10
b2
z1
z2
z3
z4
c4
y5
c6
c7
c8
c9
z3
z4
z7
z8
y8
c11
intensity
mass
0
20000
40000
60000
80000
500
1000
1500
2000
2500
AKEELEK+XL_S
A_b_ion
A_y_ion
y5+
b2+
y6++
prec
y4+
b6+
intensity
m/z
AKEELEK+XL_L
A_b_ion
A_y_ion
y5+
b2+
y6++
prec
y1+
y2+
b3+
FYSVNVDYSKLK+XL_S
B_b_ion
B_y_ion
y4+
b2+
y6+
y8+
y5+
prec
b3+
FYSVNVDYSKLK+XL_L
B_b_ion
B_y_ion
y10+
y8+
y6+
y7+
y9+
y4+
b2+
prec
y3+
b6+
b7+
b11+
m/z
